# Phrenic nerve block during nonintubated video-assisted thoracoscopic surgery: a single-centre, double-blind, randomized controlled trial

**DOI:** 10.1038/s41598-021-92003-7

**Published:** 2021-06-22

**Authors:** Yi Zhu, Guangsuo Wang, Wenli Gao, Miao Lin, Yali Li, Jiaqing Wang, Guofeng Li, Zhongliang Dai

**Affiliations:** 1 Department of Anesthesiology, Shenzhen People’s Hospital (The Second Clinical Medical College, Jinan University; The First Affiliated Hospital, Southern University of Science and Technology), Shenzhen, 518020 Guangdong Province China; 2 Department of Anesthesiology , Jingzhou Central Hospital, The Second Clinical Medical College of Yangtze University, The Jingzhou Hospital of Tongji Medical College Huazhong University of Science and Technology, Jingzhou, 434020 China; 3 Department of Thoracic Surgery, Shenzhen People’s Hospital (The Second Clinical Medical College, Jinan University; The First Affiliated Hospital, Southern University of Science and Technology), Shenzhen, 518020 Guangdong Province China

**Keywords:** Randomized controlled trials, Trauma

## Abstract

There has been interest in the use of nonintubated techniques for video-assisted thoracoscopic surgery (VATS) in both awake and sedated patients. The authors’ centre developed a nonintubated technique with spontaneous ventilation for use in a patient under general anaesthesia using a phrenic nerve block. This treatment was compared with a case-matched control group. The authors believe that this technique is beneficial for optimizing anaesthesia for patients undergoing VATS. The patients were randomly allocated (1:1) to the phrenic nerve block (PNB) group and the control group. Both groups of patients received a laryngeal mask airway (LMA) that was inserted after anaesthetic induction, which permitted spontaneous ventilation and local anaesthesia in the forms of a paravertebral nerve block, a PNB and a vagal nerve block. However, the patients in the PNB group underwent procedures with 2% lidocaine, whereas saline was used in the control group. The primary outcome included the propofol doses. Secondary outcomes included the number of propofol boluses, systolic blood pressure (SBP), pH values of arterial blood gas and lactate (LAC), length of LMA pulled out, length of hospital stay (length of time from the operation to the time of discharge) and complications after 1 month. Intraoperatively, there were increases in lactate (F = 12.31, P = 0.001) in the PNB group. There was less propofol (49.20 ± 8.73 vs. 57.20 ± 4.12, P = 0.000), fewer propofol boluses (P = 0.002), a lower pH of arterial blood gas (F = 7.98, P = 0.006) and shorter hospital stays (4.10 ± 1.39 vs. 5.40 ± 1.22, P = 0.000) in the PNB group. There were no statistically significant differences in the length of the LMA pulled out, SBP or complications after 1 month between the groups. PNB optimizes the anaesthesia of nonintubated VATS.

## Introduction

Lung cancer is the most common cancer and the leading cause of cancer-related deaths in China^1^. The deleterious effects of general anaesthesia for thoracic surgery, including numerous postoperative complications, delayed wound healing, atelectasis, pneumonia and respiratory failure, have been well described^2,3^. In 1910, Professor Jacobeus of Sweden completed the first endoscopic pleural dissection in patients with pulmonary tuberculosis, and endoscopic surgery has since been applied to thoracic surgery as a fully formed surgical technique, due to the work and research of German experts^4^. In 2003, single-port video-assisted thoracic surgery (single-port VATS) was reported to be used in the diagnosis and treatment of non-complex pleural diseases for the first time by Migliore, and its application has since been verified^5^. VATS is a feasible alternative to the use of thoracotomy, with advantages including a smaller incision, less postoperative pain, fewer complications and faster recovery^6,7^. The risks of lung injury of the dependent and nondependent lung, tracheal intubation and other complications should not be ignored, despite the use of protective ventilation strategies^8,9^.

Nonintubated general anaesthesia thoracoscopic surgery was first described by Pompeo^10,11^ and was subsequently demonstrated to be safe and feasible for various thoracic procedures^12^. The patients who have under gone nonintubated techniques were shown to spontaneously ventilate and were awake or under minimal sedation with the aid of local or regional anaesthesia^10,11,13^. It is encouraging and has been illustrated by some authors that these techniques appear to be feasible, safe and likely of greater benefit to the patient, with improved postoperative outcomes that include quicker recovery times, better pain scores, lower morbidity rates and shorter lengths of hospital stays^14,15^. However, a switch to the use of thoracotomy is unavoidable in some cases because of unsatisfactory anaesthetic and mediastinal movements during surgery^16^. Therefore, it is necessary to develop techniques for nonintubation general anaesthesia in VATS.

The phrenic nerve innervates the diaphragm, which is the major muscle of inspiration, and PNBs^16^ can theoretically inhibit mediastinal movements during VATS. Thus, the objective of this prospective study was to explore whether PNB use during nonintubated general anaesthesia VATS can decrease mediastinal movements during thoracic surgery.

## Results

### Baseline characteristics of the study participants

Eighty patients were divided into a control group and a PNB group using block randomization (1:1). Due to the that factors such as BMI, ASA score and previous histories of thoracic surgeries may potentially hamper anticipated minimally invasive procedures, we included BMI ≥ 25 kg/m^2^ and previous incidences of thoracic surgery as exclusion criteria and ASA I-II as inclusion criteria. No patients were lost or excluded after randomization and follow-up. There were no statistically significant differences between the groups concerning the baseline demographics (Table 1).

**Table 1:** Baseline demographics of the participants.

Characteristic	PNB group (n = 40)	Control group (n = 40)	P
Age (years), mean ± SD	47.30 ± 13.18	49.20 ± 12.62	0.512
Male, n (%)	18 (45.0%)	21 (52.5%)	0.502
BMI (kg/m^2^), mean ± SD	21.66 ± 2.43	21.03 ± 3.02	0.301
Operation time (min), mean ± SD	84.30 ± 5.68	86.00 ± 13.99	0.480
Snoring, n (%)	13 (32.5%)	12 (30.0%)	0.809
Mild respiratory dysfunction, n (%)	20 (50.0%)	18 (45.0%)	0.654
Increased respiratory secretions, n (%)	13 (32.5%)	15 (37.5%)	0.639
ASA I, n (%)	12 (30.0%)	10 (25.0%)	0.617
Previous thoracic surgery (%)	0 (0.0%)	0 (0.0%)	1

*BMI* body mass index, *PNB* phrenic nerve block, *SD* standard deviation.

### Perioperative outcomes

The dose of propofol that was used was significantly lower in the PNB group than in the control group (49.20 ± 8.73 vs. 57.20 ± 4.12, P = 0.000). Additionally, hospital stays were significantly shorter in the PNB group than in the control group (4.10 ± 1.39 vs. 5.40 ± 1.22, P = 0.000) (Table 2).

**Table 2:** Perioperative outcomes of the patients in the two groups.

Outcomes	PNB group (n = 40)	Control group (n = 40)	P
Propofol doses (ml), mean ± SD	49.20 ± 8.73	57.20 ± 4.12	0.000
The length of the LMA pulled out (min), mean ± SD	17.00 ± 7.58	19.60 ± 10.14	0.198
Hospital stays (days), mean ± SD	4.10 ± 1.39	5.40 ± 1.22	0.000

*PNB* phrenic nerve block, *SD* standard deviation.

The number of propofol boluses was significantly lower in the PNB group than in the control group (P = 0.002) (Table 3).

**Table 3:** The times of the propofol boluses.

The number of propofol bolus, n (%)	PNB group (n = 40)	Control group (n = 40)	P
2 or more	0	8 (20%)	0.002
1	7 (17.5%)	11 (27.5%)	
0	33 (82.5%)	21 (52.5%)	

*PNB* phrenic nerve block.

There were no statistically significant differences between the two groups in SBP (main effect, P = 0.080), although differences in the time effect and interaction effect in SBP were observed (time effect, P = 0.001; interaction effect, P = 0.001). The pH of arterial blood gas (main effect, P = 0.006) was lower and of lactate (main effect, P = 0.001) was higher in the PNB group over time, with both groups (in regards to pH values of arterial blood gas and lactate) having significant differences over time (time effect, P = 0.000 for both groups). Additionally, the interaction effect in the pH of arterial blood gas exhibited no significant differences between the two groups (interaction effect, P = 0.136), and the interaction effect in lactate exhibited no significant differences between the two groups (interaction effect, P = 0.076) (Table 4).

**Table 4:** SBP, arterial pH and blood lactate measured at three different perioperative time points.

Variable	PNB group	Control group	Main effect P value	Time effect P value	Interaction effect P value
	(n = 40)	(n = 40)			
**SBP (mmHg), mean ± SD**					
The time the thoracoscope was inserted	118.20 ± 17.16	111.60 ± 15.19	0.08	0.001	0.001
The time the thoracoscope was pulled out	107.80 ± 16.73	111.60 ± 14.67			
The time the LMA was pulled out	112.90 ± 13.14	101.80 ± 16.31			
**The pH of arterial blood gas, mean ± SD**					
The time the thoracoscope was inserted	7.33 ± 0.07	7.35 ± 0.02	0.006	0.000	0.136
The time the thoracoscope was pulled out	7.25 ± 0.04	7.26 ± 0.03			
The time the LMA was pulled out	7.30 ± 0.03	7.33 ± 0.02			
**Lactate (mmol/l), mean ± SD**					
The time the thoracoscope was inserted	1.25 ± 0.78	0.82 ± 0.33	0.001	0.000	0.076
The time the thoracoscope was pulled ou	0.82 ± 0.46	0.61 ± 0.28			
The time the LMA was pulled out	0.86 ± 0.43	0.60 ± 0.19			

*PNB* phrenic nerve block, *SD* standard deviation, *SBP* systolic blood pressure.

### Postoperative complications after 1 month

The main postoperative complications that were encountered during the follow-up period were pulmonary air leakage, pneumoderma and pneumothorax, but these occurred with low incidences. There were no statistically significant differences between the groups in the incidences of pulmonary air leakage, pneumoderma and pneumothorax (Table 5).

**Table 5:** Postoperative complications of the patients in the two groups.

Complication, n (%)	PNB group (n = 40)	Control group (n = 40)	P
Pulmonary air leakage	3 (7.5%)	2 (5.0%)	1.000
Pneumoderma	2 (5.0%)	2 (5.0%)	1.000
Pneumothorax	2 (5.0%)	1 (2.5%)	1.000

*PNB* phrenic nerve block.

## Discussion

Numerous previous clinical investigations have shown that thoracoscopic surgery under epidural anaesthesia is safe and effective. There are many advantages of nonintubation general anaesthesia under epidural anaesthesia, including a shorter operation time, a shorter postoperative fasting time, less antibiotic use, shorter hospital stays, decreased nursing demand and higher patient satisfaction^10,11,13–15^. Other scholars believe that nonintubated anaesthesia with the aid of paravertebral or intercostal nerve blocks is feasible and safe for thoracoscopic surgery, and nonintubated general anaesthesia under epidural anaesthesia and regional block is a feasible alternative to intubated general anaesthesia^17–19^. There are also several additional benefits, such as a quicker induction of anaesthesia, improved intraoperative haemodynamic stability, reduced intraoperative blood loss, a shorter operation time, a shorter duration of chest tube drainage and a shorter hospital stay for the patients^20^.

A notable finding of our study is that the dose of propofol that was administered for anaesthesia was significantly lower in the PNB group, thus indicating that it is beneficial for optimizing nonintubated general anaesthesia in VATS by using PNB. PNB was also associated with a faster recovery time and a shorter hospital stay, which likely resulted from the lower dose of propofol that was administered. We speculate that the use of PNB in the patients helped to reduce cough during surgery, which led to a lower dose of propofol that needed to be administered.

In our study, severe mediastinal movements were not encountered during surgery. Lung isolation was achieved with spontaneous deflation of the nondependent lung, due to the iatrogenic pneumothorax that was induced during the opening of the pleura. Subsequently, the surgeon used an electric knife, titanium clip and disposable stapler to separate the tissues, after which the surgeon clamped the blood vessels and cut off the diseased lung tissue. After resection, it was necessary to test whether there was air leakage into the lung. The wound was covered with anti-adhesion material after haemostasis. The use of titanium clips and disposable staplers is necessary, which results in a faster surgical process, less bleeding and decreased risks of injury for the patients. The possibility of postoperative pulmonary complications can be reduced by testing for pulmonary air leakage and using anti-adhesion materials. However, it is difficult for VATS to achieve satisfactory results in complex surgical conditions, such as severe adhesion of the lung tissue or of the bullae of lung. During the operation, there are often accidental injuries to blood vessels and nerves, as well as the excessive resection of lung tissue, which are often accompanied by intraoperative adverse events, such as massive blood loss, multiple incidences of pneumothorax, difficult recovery of postoperative lung function and even death. Once these conditions occur, surgeons need to consider the possibility of thoracotomy and the possibility of stopping the study at any time. A fall in blood pressure is known to occur during propofol-based anaesthesia^21^, and PNB has a transient effect on pulmonary function that is limited to the duration of the operation^22,23^. In the present study, all of these factors were indeed observed in both groups. However, there were no significant differences between the groups in regard to SBP, and the pH of arterial blood gas was lower and of lactate was higher in the PNB group over time. Additionally, there were no significant differences between the groups in the incidences of pulmonary complications, which indicated that PNB had no significant adverse reactions to the patients.

There were some limitations in this study. First, due to the fact that this was a single-centre study, the generalizability of the findings is not known. Second, the sample size was quite small. Thus, the study may have been underpowered for detecting actual differences between the groups. In addition, there was the possibility of bias in the baseline characteristics between groups due to the sample size, although no differences were detected in the baseline demographics. Finally, the follow-up period was short, and longer-term outcomes were not assessed. Large-scale, multicentre, randomized controlled trials are required to extend our findings.

In conclusion, the use of PNB can reduce the dose of anaesthetic during nonintubated VATS and improve postoperative recovery without the occurrence of additional complications.

## Methods

### Study design and participants

This single-centre, double-blind, randomized controlled trial recruited patients requiring thoracoscopic surgery at Shenzhen People’s Hospital (Guangdong, China) between September 2018 and March 2019. All of the patients who were recruited in this study were fully informed of the advantages and disadvantages of participating in this study before they agreed to participate. After obtaining the informed consent of the patients, they were incorporated into the study. Patients who failed to provide informed consent were excluded from this study.

**Figure 1:**
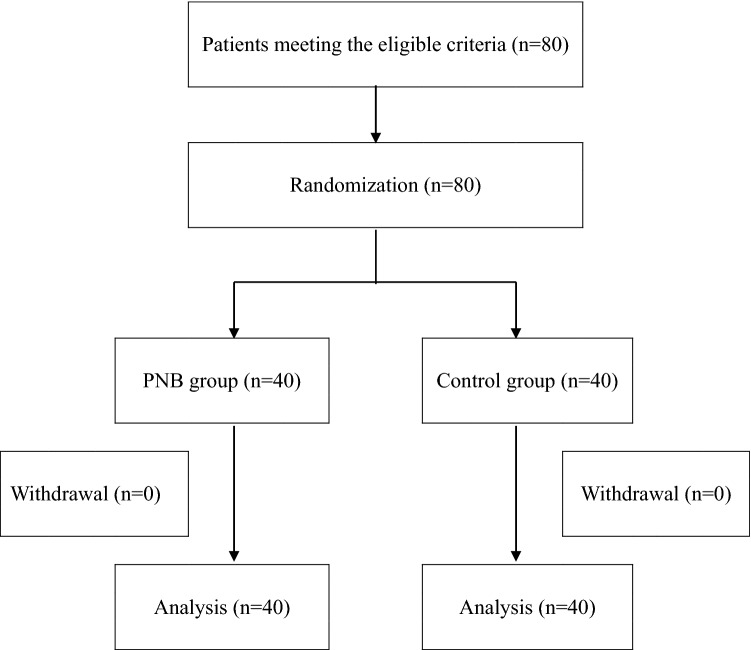
Study flowchart.

Each patient selected an envelope that used the random number table method to determine their specific grouping information, and the patients were divided into a control group and a PNB group using block randomization (1:1). The study flowchart in described in Fig. 1. The inclusion criteria were as follows: (1) ages of 18–65-years-old; (2) no contraindications of general anaesthesia and local anaesthesia; (3) ASA I-II; (4) a straightforward operation without incidences of major surgical trauma was anticipated (e.g., the removal of a tumour at a single surgical site without invasions of the peripheral vessels and nerves and no requirement for the use of multidisciplinary surgery); (5) greater risks of general anaesthesia being associated with tracheal intubation (e.g., the pulmonary bulla); and (6) no obvious cardiopulmonary dysfunction. The exclusion criteria were as follows: (1) refusal to participate; (2) Mallampati score ≥ 3; (3) body mass index (BMI) ≥ 25 kg/m^2^; (4) haemodynamic instability; (5) international normalized ratio (INR) ≥ 1.5; (6) incidences of respiratory infection, persistent cough or hypersecretion of airway mucus, with a high risk of reflux; (7) previous histories of thoracic surgery or extensive thoracic adhesions; (8) hypoxemia (PaO_2_ [alveolar oxygen partial pressure] < 60 mmHg) and/or hypercapnia (PaCO_2_ [arterial partial pressure of carbon dioxide] > 50 mmHg); (9) high intracranial pressure; (10) requirements of pulmonary isolation techniques; (11) inexperienced or poorly cooperated surgical teams; (12) contralateral phrenic paralysis; (13) incidences of neurological or psychiatric disorders, such as severe anxiety and epilepsy; and (14) central line lesion and resection range > 6 cm.

During the operation, the study was planned to be stopped or changed to the use of an endotracheal intubation if adverse events or refusals of the patients occurred.

- Adverse events were defined as events that were not planned to affect the operation, including: patient being unable to tolerate the stimulation of the operation; the effect of non-intubation anaesthesia being unable to meet the needs of the surgery and lacking improvement when the anaesthesia depth was adjusted; refractory hypoxemia being observed (blood oxygen less than 90% for more than 5 min); the blood pressure being decreased by 30%, compared to the preoperative blood pressure; severe bleeding that did not improve with adjustments in anaesthesia; and risks of death. Serious adverse events were defined as incidences of refractory severe hypoxemia, severe bleeding (even during shock), errors in inhalation and other events.
- During the operation, anaesthesiologists and surgeons jointly monitored the patient's vital signs to ensure an optimal operation and the safety of the patients.
- Anaesthesiologists planned to identify and report any adverse events. In addition, the data collector aimed to document any adverse events.
- Anaesthesiologists, surgeons and data collectors reviewed the summarized safety information after every 5 patient operations.
- The stopping rules regarded the incidences of adverse events or the refusals of the patients.

The Ethics Committee of Shenzhen People’s Hospital approved this study (No. 2018-129). This study was registered at clinicaltrials.gov (NCT03653494), and the first registration date was 08/31/2018. All of the methods of this study were performed in accordance with the relevant guidelines of the ethics committee of Shenzhen People's Hospital and clinicaltrials.gov. During this study, informed consent was obtained from the patients, and the privacy and interests of the patients were not infringed.

### Interventions

The patients, anaesthesiologists and surgeons were blinded to the allocation of the patients, and the treatments applied to the phrenic nerve block (lidocaine or saline) were prepared by investigators who did not participate in the operation.

The same primary anaesthesiologist and surgeon were present at all of the operations, and all of the anaesthesiologists and surgeons who were involved in the procedures had abundant operational experience and relevant qualifications. The patients in both groups received nonintubated general anaesthesia, paravertebral nerve block, PNB and vagal nerve block treatments. General anaesthesia was administered using propofol, remifentanil and dexmedetomidine to maintain a bispectral index (BIS) of 45–60. Twenty millilitres of 0.5% ropivacaine was used for the paravertebral nerve block at T_5–6_ and T_7-8_. Ropivacaine (0.75%, 2.5 mL) and lidocaine (2%, 2.5 mL) were used for the intrathoracic vagal nerve block. In addition, lidocaine (2%, 5 mL) was used for the patients in the PNB group who underwent PNB on the operative side, and sodium chloride (0.9%, 5 mL) was used in the control group. All of the drugs and equipment related to anaesthesia and surgery were consistently used during the study period.

### Follow-up and outcome measures

The patients were followed-up for 1 month. The primary outcome measure was the propofol doses for general anaesthesia. The secondary outcome measures were as follows: (1) the number of propofol boluses; (2) SBP and pH values of arterial blood gas and lactate being measured at the time when the thoracoscope was inserted, as well as at the time when the thoracoscope was pulled out and the length of the LMA pulled out; (3) the length of the LMA pulled out was measured from the end of the operation to the time when the LMA was pulled out; (4) hospital stays; and (5) postoperative complications, including postoperative pulmonary complications at 1 month after surgery.

### Statistical analysis

The data were analysed by using SPSS 19.0 (IBM SPSS). According to the results of a preliminary study, the calculated sample size was n_1_ = n_2_ = 33. Assuming a loss to follow-up of 20%, each group was required to include 40 patients at enrolment.

The categorical variables are expressed as frequencies and percentages, and comparisons of the ratios were performed by using the chi-squared test or Fisher’s exact test. The continuous variables were tested for normality. Normally distributed continuous variables are expressed as the mean ± SD and were compared between the two groups using Student’s t-test. Nonnormally distributed continuous variables are presented as medians (ranges) and were compared between groups using the Mann–Whitney U test. Systolic blood pressure and the pH values of arterial blood gas and lactate at the three time points were analysed using a two-way repeated measures analysis of variance (ANOVA). The times of intraoperative propofol bolus administration were analysed using the Wilcoxon test. P < 0.05 was considered statistically significant.
